# A 4K score/MRI‐based nomogram for predicting prostate cancer, clinically significant prostate cancer, and unfavorable prostate cancer

**DOI:** 10.1002/cnr2.1357

**Published:** 2021-03-04

**Authors:** Vinayak G. Wagaskar, Stanislaw Sobotka, Parita Ratnani, James Young, Anna Lantz, Sneha Parekh, Ugo Giovanni Falagario, Li Li, Sara Lewis, Kenneth Haines, Sanoj Punnen, Peter Wiklund, Ash Tewari

**Affiliations:** ^1^ Department of Urology Icahn School of Medicine at Mount Sinai Hospital New York New York USA; ^2^ Department of Pathology Icahn School of Medicine at Mount Sinai Hospital New York New York USA; ^3^ Department of Radiology Icahn School of Medicine at Mount Sinai Hospital New York New York USA; ^4^ Department of Urology University of Miami, Miller School of Medicine Miami Florida USA

**Keywords:** 4K score test, biopsy, multiparametric MRI, prostate cancer

## Abstract

**Background:**

The detection of prostate cancer requires histological confirmation in biopsy core. Currently, number of unnecessary prostate biopsies are being performed in the United States. This is due to the absence of appropriate biopsy decision‐making protocol.

**Aim:**

To develop and validate a 4K score/multiparametric magnetic resonance imaging (mpMRI)‐based nomogram to predict prostate cancer (PCa), clinically significant prostate cancer (csPCa), and unfavorable prostate cancer (uPCa).

**Methods and Results:**

Retrospective, single‐center study evaluating a cohort of 574 men with 4K score test >7% or suspicious digital rectal examination (DRE) or Prostate Imaging Reporting and Data System (PI‐RADS) scores 3, 4, or 5 on mpMRI that underwent systematic and/or mpMRI/ultrasound fusion–targeted prostate biopsy between 2016 and 2020. External cohort included 622 men. csPCa and uPCa were defined as Gleason score ≥3 + 4 and ≥4 + 3 on biopsy, respectively. Multivariable logistic regression analysis was performed to build nomogram for predicting PCa, csPCa, and uPCa. Validation was performed by plotting the area under the curve (AUC) and comparing nomogram‐predicted probabilities with actual rates of PCa, csPCa, and uPCa probabilities in the external cohort.

4K score, a PI‐RADS ≥4, prostate volume and prior negative biopsy were significant predictors of PCa, csPCa, and uPCa. AUCs were 0.84, 0.88, and 0.86 for the prediction of PCa, csPCa, and uPCa, respectively. The predicted and actual rates of PCa, csPCa, and uPCa showed agreement across all percentage probability ranges in the validation cohort. Using the prediction model at threshold of 30, 30% of overall biopsies, 41% of benign biopsies, and 19% of diagnosed indolent PCa could be avoided, while missing 9% of csPCa.

**Conclusion:**

This novel nomogram would reduce unnecessary prostate biopsies and decrease detection of clinically insignificant PCa.

## 1 BACKGROUND

Prostate cancer is the most common cancer in men in the United States, accounting for an estimated 19% of all newly diagnosed cancers in 2018.^1^ Prostate‐specific antigen (PSA) is the only molecular marker routinely used for detection of prostate cancer (PCa), and screening with PSA has been shown to reduce prostate cancer mortality.^2^ However, numerous studies have demonstrated the diagnostic limitations of PSA resulting in overdiagnosis of indolent cancers, frequent unnecessary prostate biopsies, the results of which are benign, and overtreatment with significant morbidity.^3^ Alternative strategies for the detection of significant PCa are needed to avoid potentially morbid, invasive procedures in men who are unlikely to be diagnosed with prostate cancer.

4K score test (OPKO Diagnostics, Woburn, MA) is a serum biomarker–based test on a four‐kallikrein panel including kallikrein‐related peptidase 2 (hK2), intact PSA, free PSA, and total PSA, as well as incorporating clinical information such as biopsy history and DRE findings. The 4K score test has repeatedly been shown to predict prostate cancer (PCa) biopsy outcome in men with elevated PSAs while also significantly reducing the number of unnecessary biopsies.^4^, ^5^, ^6^ Furthermore, multiparametric magnetic resonance imaging (mpMRI) in combination with targeted biopsies has emerged as an effective tool to detect clinically significant disease.^7^, ^8^, ^9^ Studies combining biomarkers and MRI‐targeted biopsies for prostate cancer detection have also been proven to decrease the number of biopsies and avoid overdetection of indolent cancer to an even greater extent.^10^

Although the mpMRI and the 4K score test are both used in American clinical practice for the evaluation of prostate cancer, there are no reports on the impact of using these tests in combination. We hypothesize that both of these tests will provide independent and complementary value, and when combined, will improve the detection of clinically significant disease compared to either test alone.^11^ The aim of this study was to develop a nomogram using 4K score test and mpMRI to detect clinically significant prostate cancer (csPCa) in men with an elevated PSA and/or abnormal DRE.

## 2 MATERIAL AND METHODS

### 2.1 Study population

We retrospectively reviewed our institution's prostate biopsy database to extract relevant patient records. A total of 1100 patients underwent 4K score test between January 2016 and April 2020. Of these, 584 (53%) underwent systematic or combined systematic and MRI/transrectal ultrasound (TRUS) fusion biopsy by a single expert surgeon (A.K.T. ) with 20 years of experience. Indications of biopsy were 4K score test of >7%, suspicious DRE, PI‐RADS scores of 3, 4 or 5 on mpMRI, or combination of any of the above. Men who have contraindication for prebiopsy mpMRI (n = 10) were excluded. In total, 574 men were used to build the model (development cohort). For validation, we used a cohort of 622 men that underwent systematic or combined systematic and MRI/TRUS fusion biopsy for similar indications (4K score test >7%, suspicious DRE, PI‐RADS scores of 3, 4, or 5 on mpMRI) at the University of Miami.

All men underwent standardized 3‐Tesla mpMRI prior to prostate biopsy. All mpMRI examinations were compliant with the American College of Radiology recommendations for technical specifications. All mpMRI results were evaluated according to PI‐RADS Version 2 (PI‐RADS V2) by clinical radiologists with experience in prostate imaging.

All biopsies were performed using a spring‐loaded biopsy gun and 18‐gauge needles. For targeted biopsies, Artemis MRI/TRUS fusion device (Innomedicus, Cham, Switzerland) was used and extra 2‐4 cores taken from each lesion in addition to standard 12 cores biopsy. An experienced genitourinary pathologist (K.H. III) reviewed all biopsy samples.

### 2.2 Outcomes definition and statistical analysis

The outcome for predicting PCa was defined as a Gleason score of ≥3 + 3 on biopsy, and men with this outcome were considered cases. Men with negative biopsy were considered controls. The outcome for predicting csPCa was defined as a Gleason score of ≥3 + 4 on biopsy; men with this outcome were considered cases, and the remaining men with negative biopsy and men with Gleason score 3 + 3 on biopsy were considered controls. The outcome for predicting uPCa was defined as a Gleason score of ≥4 + 3 on biopsy; men with this outcome were considered cases, while the remaining men with negative biopsy, Gleason score 3 + 3, 3 + 4, were considered controls.

Descriptive statistics for the two groups were calculated. Continuous variables were reported as median and interquartile range (IQR) and compared using a Mann–Whitney *U* test. Categorical variables were reported as rates and were tested with a chi‐square test, as appropriate. The prediction model included the following variables: age, family history of prostate cancer, history of negative prior biopsy, 4K score test, DRE findings, mpMRI prostate volume, and highest PI‐RADS score. We are aware that 4K score test incorporates clinical parameters like age, family history, DRE, and prior biopsy history. Therefore, we calculated matrix of correlation coefficients between 4K score test and these predictors. We also ran variance inflation factor analysis (the inflation in the variances of the parameter estimates due to collinearities among predictors) to evaluate the potential presence of substantial multicollinearity between these predictors in our model.

Analysis of correlation coefficients between predictors as well as variance inflation index of predictors did not indicate presence of strong collinearity between 4K and other predictors in our model. There were no strong correlations (>0.8) between 4K score test and other predictors. PI‐RADS scores of 1 and 2 were grouped for the purpose of analysis. Multivariable binary logistic regression analysis was performed for presence of PCa, csPCa, and uPCa in the development cohort. The nomogram predicting PCa, csPCa, and uPCa was built based on coefficients of the logit function.

Nomogram validation was performed in the external cohort in two stages. First, receiver‐operating characteristics (ROCs) were plotted for presence of PCa, csPCa, and uPCa using the same variables that were used to build the nomogram. Second, calibration graphs were plotted by grouping sorted nomogram‐predicted probabilities from the development cohort into deciles and then comparing the mean prediction of each group with the observed proportion of men from the validation cohort with PCa or csPCa or uPCa. Using nomogram‐derived probability cut‐offs, we calculated the number of biopsies that could be avoided without missing PCa, csPCa, or uPCa in the validation cohort. Decision curve analysis (DCA) was performed to evaluate the performance of the prediction models in the validation cohort. Statistical analyses were performed using STATA 12 (StataCorp LP, College Station, TX, USA) and SAS 9.4 (SAS Institute, Cary, NC, USA). All tests were two‐tailed with a significance level of *P* < .05.

## 3 RESULTS

Of the total 574 men in the development cohort, 232 (40%) were diagnosed with PCa, while the remaining 342 men (60%) were not having PCa. Median age was 66 years [interquartile range (IQR) 60, 70] and 64 years (IQR 59, 69) for PCa and benign biopsies, respectively; median PSA was 6.1 ng/mL (IQR 4.5, 7.9), 5.3 ng/mL (IQR 3.4, 7.7); and median 4K score test was 28 (IQR 13, 55), 9 (IQR 3, 18) for men with PCa and men with benign biopsy, respectively. Of the 232 men with PCa, 89 (38%), 65 (28%), 27 (12%), 30 (13%), and 21 (9%) had Gleason scores of 3 + 3, 3 + 4, 4 + 3, 4 + 4, and 4 + 5/5 + 4/5 + 5, respectively. Of the 622 men in the validation cohort, 328 (53%) did not have prostate cancer on biopsy. Of the remaining 294 who had cancer in the validation cohort, 121 (41%), 77 (26%), 29 (10%), 29 (10%), and 38 (13%) had Gleason scores of 3 + 3, 3 + 4, 4 + 3, 4 + 4, and 4 + 5/5 + 4/5 + 5, respectively (Table 1).

**TABLE 1:** Comparison of factors between cases and controls in development and validation cohort

Mount Sinai (Development) Cohort				University of Miami (Validation) Cohort		
Factors	Benign biopsies n = 342 (60%)	Prostate cancer n = 232 (40%)	*P* value	Benign biopsies N = 328 (53%)	Prostate cancer N = 294 (47%)	*P* value
Age years (Median, IQR)	64 (59, 69)	66 (60, 70)	.0339	61 (59, 69)	60 (58,70)	.7746
PSA ng/mL (Median, IQR)	5.3 (3.4, 7.7)	6.1 (4.5, 7.9)	.001	5.6 (3.9, 8.6)	6.0 (4.5, 8.4)	.0655
4K score test (OPKO Diagnostics, Woburn, MA) (Median, IQR)	9 (3,18)	28 (13, 55)	<.0001	11 (4, 21)	28 (13, 56)	<.0001
Prior negative biopsy			<.0001			<.0001
No	191 (56%)	196 (85%)		191 (58%)	221 (75%)	
Yes	151 (44%)	36 (15%)		137 (42%)	73 (25%)	
DRE			<.0001			.0358
Normal	250 (73%)	127 (55%)		250 (76%)	202 (69%)	
Suspicious	92 (27%)	105 (45%)		78 (24%)	92 (31%)	
MRI lesion highest PI‐RADS score			<.0001			<.0001
0–2	194 (57%)	50 (21%)		144 (44%)	38 (13%)	
3	68 (20%)	29 (13%)		102 (31%)	51 (17%)	
4	70 (20%)	94 (41%)		72 (22%)	136 (46%)	
5	10 (3%)	59 (25%)		10 (3%)	69 (24%)	
Prostate volume cc (Median, IQR)	60 (43, 82)	41 (30, 56)	<.0001	60 (42, 83)	41 (31,57)	<.0001
Biopsy						
Systematic	201 (59%)	71 (31%)		144 (44%)	38 (13%)	
Systematic and targeted	141 (41%)	161 (69%)		184 (56%)	256 (87%)	
Biopsy GS						
0	342 (100%)	0		328 (100%)	0	
3 + 3	0	89 (38%)		0	121 (41%)	
3 + 4	0	65 (28%)		0	77 (26%)	
4 + 3	0	27 (12%)		0	29 (10%)	
4 + 4/3 + 5/5 + 3	0	30 (13%)		0	29 (10%)	
4 + 5/5 + 4/5 + 5	0	21 (9%)		0	38 (13%)	

Abbreviations: DRE, digital rectal examination; GS, Gleason score; IQR, interquartile range; MRI, magnetic resonance imaging; PI‐RADS, prostate imaging reporting and data system version 2; PNB, prior negative biopsy; PSA, prostate‐specific antigen.

### 3.1 Univariable and multivariable analysis predicting PCa, csPCa, and uPCa

In univariate analysis, 4K score test, family history of prostate cancer, prior negative biopsy, DRE findings, and PI‐RADS 3, 4, 5, and MRI prostate volume emerged as significant predictors of PCa, csPCa, and uPCa. In multivariate analysis, 4K score test, PI‐RADS scores of 4 and 5, prostate volume, and history of prior negative biopsy were significantly associated with PCa, csPCa, and uPCa (all *P* < .05), while family history of PCa was significant in predicting PCa and uPCa; PI‐RADS score 3 was found significant predictor for PCa (Table 2).

**TABLE 2:** The results of multivariable analysis

Variable	Odds Ratio	SE	*P* value	95% Conf. interval for OR	
Predicting presence of PCa					
Age	1.018	0.015	.210	0.998	1.049
4K score test (OPKO Diagnostics, Woburn, MA)	1.033	0.006	.000	1.020	1.047
DRE	0.971	0.226	.900	0.614	1.535
Prior negative biopsy	0.492	0.125	.006	0.298	0.813
Prostate volume	0.978	0.004	.000	0.970	0.987
MRI PI‐RADS score					
3	2.114	0.641	.014	1.166	3.831
4	4.402	1.106	.000	2.690	7.203
5	9.235	4.028	.000	3.927	21.716
Predicting presence of csPCa					
Age	1.021	0.019	.267	0.983	1.060
4K score test (OPKO Diagnostics, Woburn, MA)	1.033	0.006	.000	1.020	1.047
DRE	1.397	0.381	.220	0.818	2.386
Prior negative biopsy	0.288	0.111	.001	0.135	0.616
Prostate volume	0.977	0.054	.000	0.967	0.988
MRI PI‐RADS score					
3	2.650	1.187	.030	1.102	6.378
4	8.366	2.894	.000	4.246	16.483
5	18.702	8.512	.000	7.664	45.639
Predicting presence of uPCa					
Age	1.072	0.024	.002	1.026	1.119
4K score test (OPKO Diagnostics, Woburn, MA)	1.024	0.007	.000	1.011	1.038
DRE	1.472	0.463	.219	0.794	2.727
Prior negative biopsy	0.189	0.109	.004	0.060	0.587
Prostate volume	0.994	0.004	.211	0.985	1.003
MRI PI‐RADS score					
3	1.767	1.196	.401	0.468	6.666
4	7.338	3.531	.000	2.856	18.846
5	13.670	7.462	.000	4.689	39.849

Abbreviations: csPCa, clinically significant prostate cancer; DRE, digital rectal examination finding; PCa, prostate cancer; PI‐RADS, Prostate Imaging Reporting and Data System; uPCa, unfavorable prostate cancer.

### 3.2 Nomogram to estimate risk of PCa, csPCa, and uPCa

**FIGURE 1:**
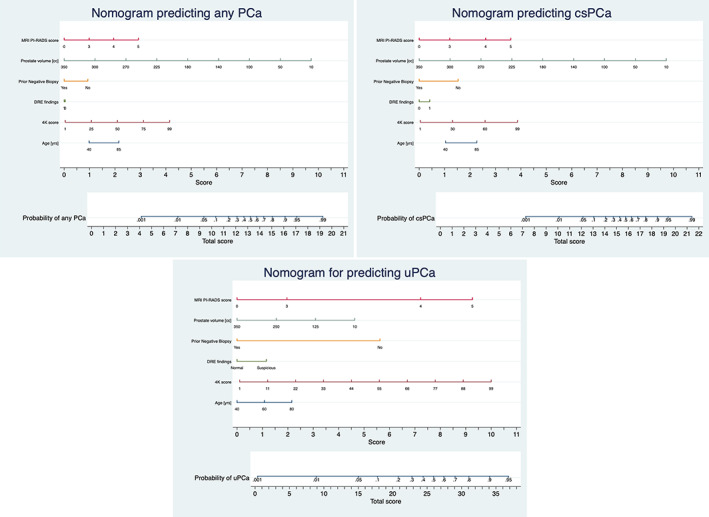
Nomogram prediction model for predicting prostate cancer, clinically significant prostate cancer and unfavourable prostate cancer at the time of biopsy. Steps for assessing cancer probability from the nomogram are as follows: 1. Locate the patient's variable Age on corresponding axis. 2. Draw a line straight download to the score axis to determine how many points towards the probability of cancer the patient is scored for his Age. 3. Repeat the process for each additional variable [ 4K score test, prior negative biopsy, DRE, MRI Prostate volume and MRI PI‐RADS score]. 4. Total the points for each of the predictors. 5. Locate the final sum on the total score axis. 6. Draw a line straight up to find the patient's probability of having PCa, csPCa and uPCa. Total scores correspond to a probability value for PCa, csPCa and uPCa. Abbreviations: PCa‐ prostate cancer, csPCa‐ clinically significant prostate cancer, uPCa‐unfavourable PCa DRE‐ digital rectal examination finding, PI‐RADS‐ Prostate Imaging Reporting and Data System

Figure 1A‐C illustrate the nomogram developed for prediction of PCa, csPCa, and unfavorable PCa, respectively, in the development cohort. MRI PI‐RADS score, 4K score test, prostate volume, and history of prior negative biopsy were significantly contributing to the total score that eventually determined nomogram probability of PCa, csPCa, and unfavorable PCa.

### 3.3 Nomogram validation

**FIGURE 2:**
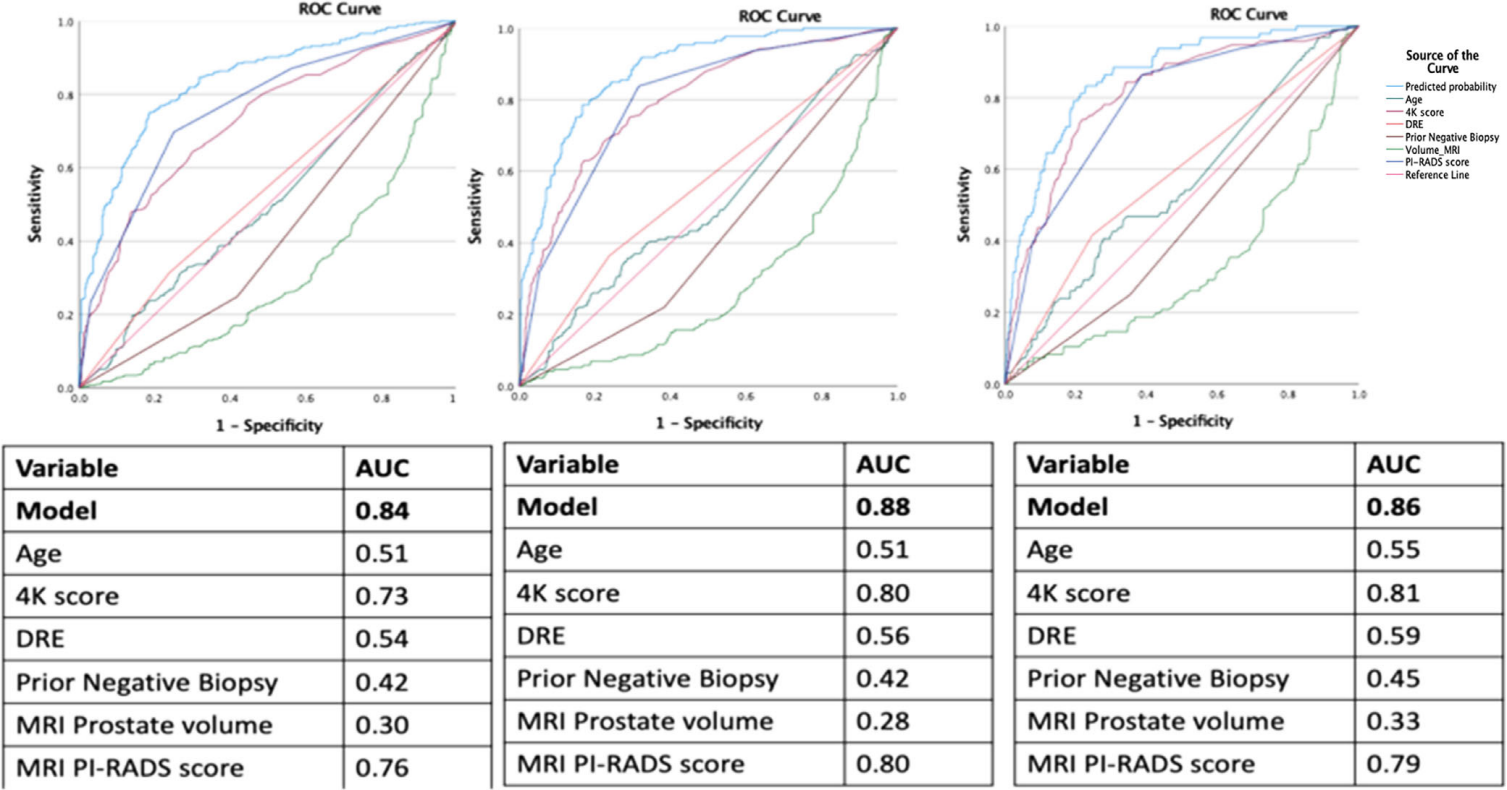
Area under curve characteristics for predicting PCa, csPCa, and uPCa

Area under receiver operating curves (AUCs) for predicting PCa, csPCa, and unfavorable PCa in the validation cohort were 0.84. 0.88, and 0.86, respectively (Figure 2). 4K score test and mpMRI PI‐RADS score showed significant contribution for building AUCs.

**FIGURE 3:**
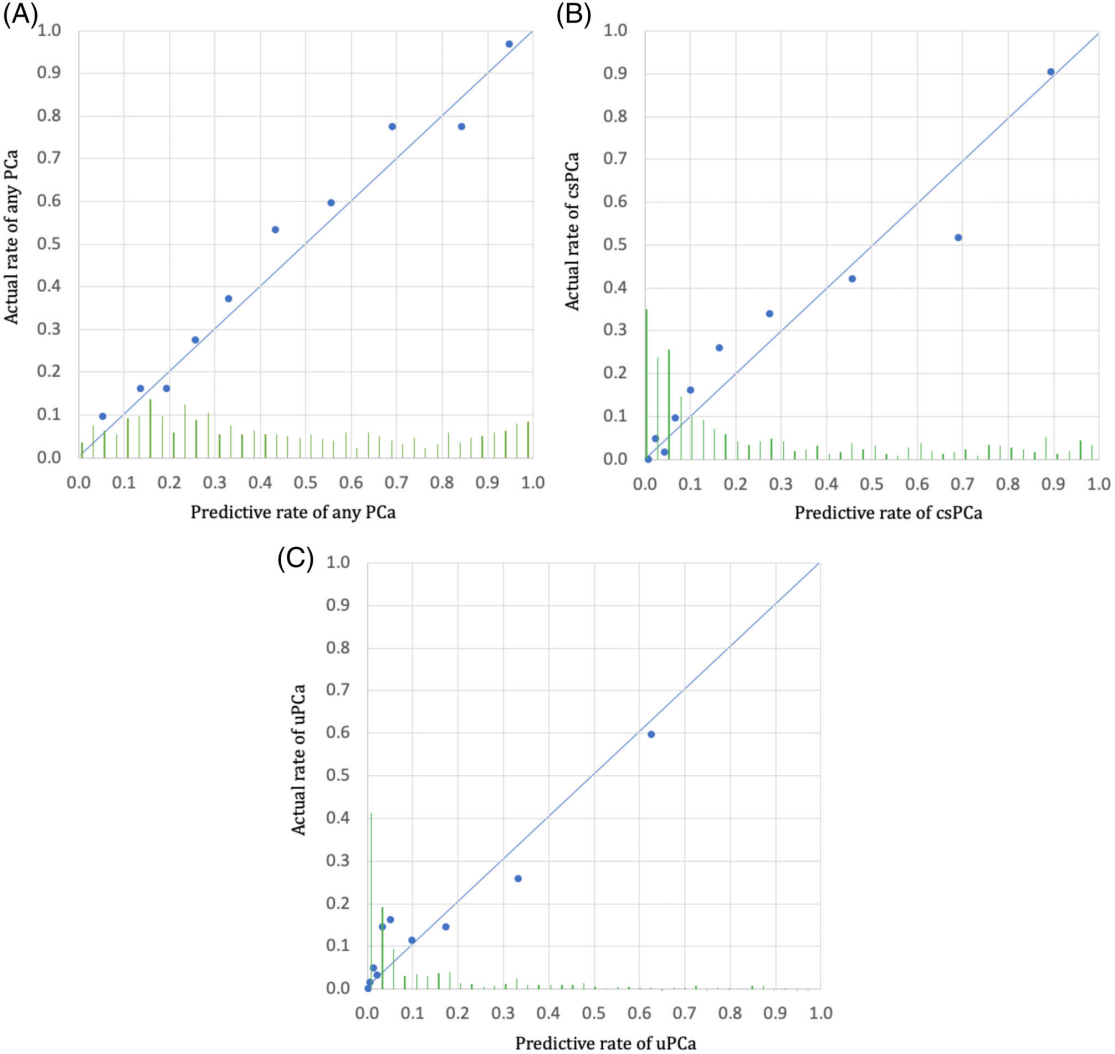
A, Predictive probabilities of cancer for each case in the testing cohort are sorted by probability of PCa calculated from the training model, respectively. Each point (average of 60 subsequent cases) illustrates the comparison between predictive probability (calculated from the training model) and actual cancer rate for this group of cases. Points on the diagonal line (0, 0 and 1, 1), show the agreement between the predicted and actual rate of PCa and the validated training model. The histogram of the calculated probabilities for the validation cohort is shown along the horizontal axis. B, Predictive probabilities of cancer for each case in the testing cohort are sorted by probability of csPCa calculated from the training model, respectively. Each point (average of 60 subsequent cases) illustrates the comparison between predictive probability (calculated from the training model) and actual cancer rate for this group of cases. Points on the diagonal line (0, 0 and 1, 1) show the agreement between the predicted and actual rate of csPCa and the validated training model. The histogram of the calculated probabilities for the validation cohort is shown along the horizontal axis. C, Predictive probabilities of cancer for each case in the testing cohort are sorted by probability of uPCa calculated from the training model, respectively. Each point (average of 60 subsequent cases) illustrates the comparison between predictive probability (calculated from the training model) and actual cancer rate for this group of cases. Points on the diagonal line (0, 0 and 1, 1) show the agreement between the predicted and actual rate of uPCa and the validated training model. The histogram of the calculated probabilities for the validation cohort is shown along the horizontal axis

We evaluated the nomogram's calibration by comparing predicted and actual probabilities of PCa, csPCa, and unfavorable PCa in the validation cohort. There was agreement between the predicted and actual rate of probabilities (0%‐100%) for PCa, csPCa, and unfavorable PCa as seen by points on the diagonal line in Figure 3A‐C, respectively.

**FIGURE 4:**
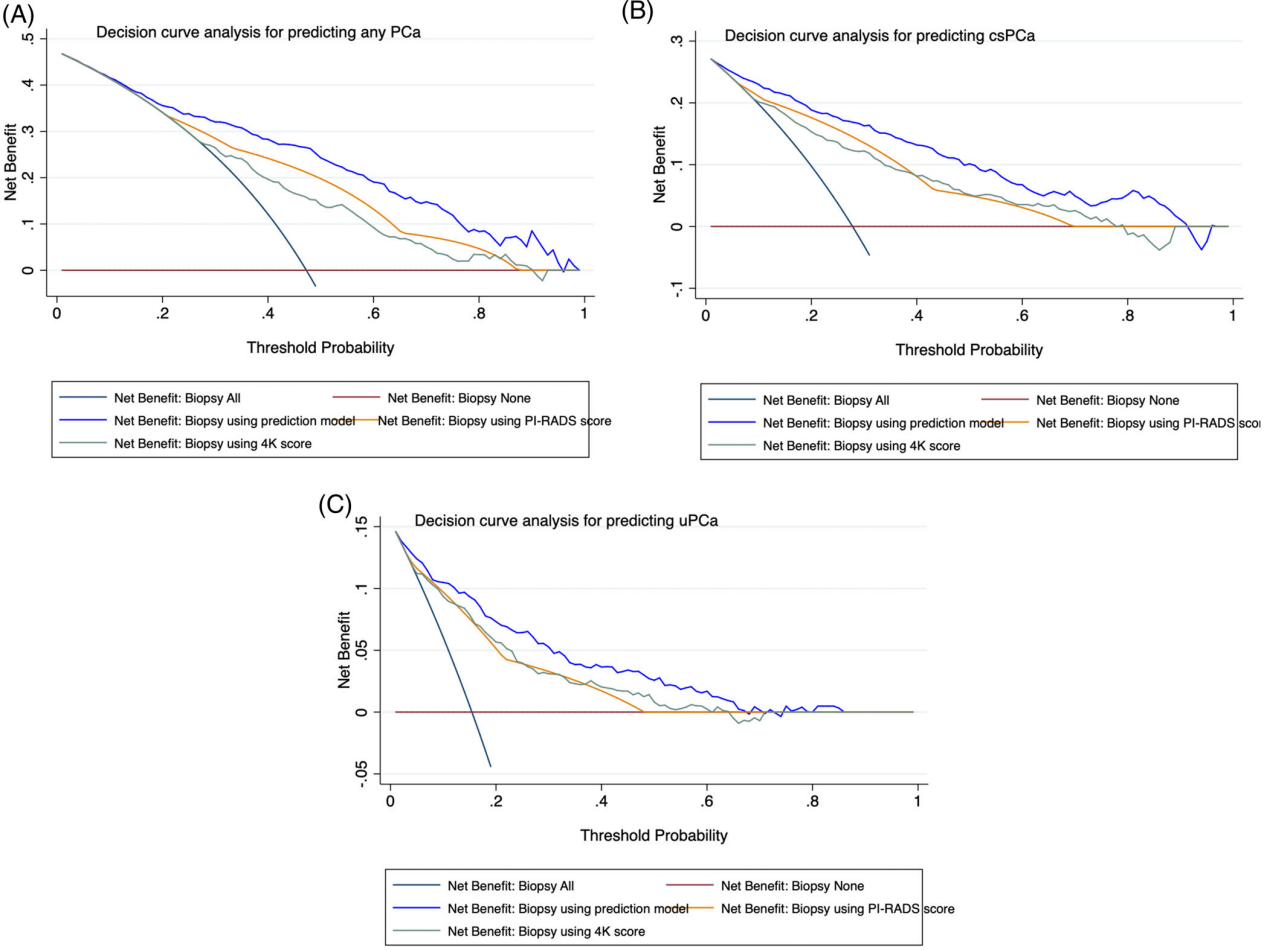
A, Decision curve analysis for predicting PCa using model vs using 4K score or PI‐RADS score alone. B, Decision curve analysis for predicting csPCa using model vs using 4K score or PI‐RADS score alone. C, Decision curve analysis for predicting uPCa using model vs using 4K score or PI‐RADS score alone

DCA showed superior clinical risk prediction for 5%‐95% of nomogram‐derived probabilities for predicting PCa, csPCa, and uPCa than relying on 4K score test alone or PI‐RADS score alone. (Figure 4A‐C).

**FIGURE 5:**
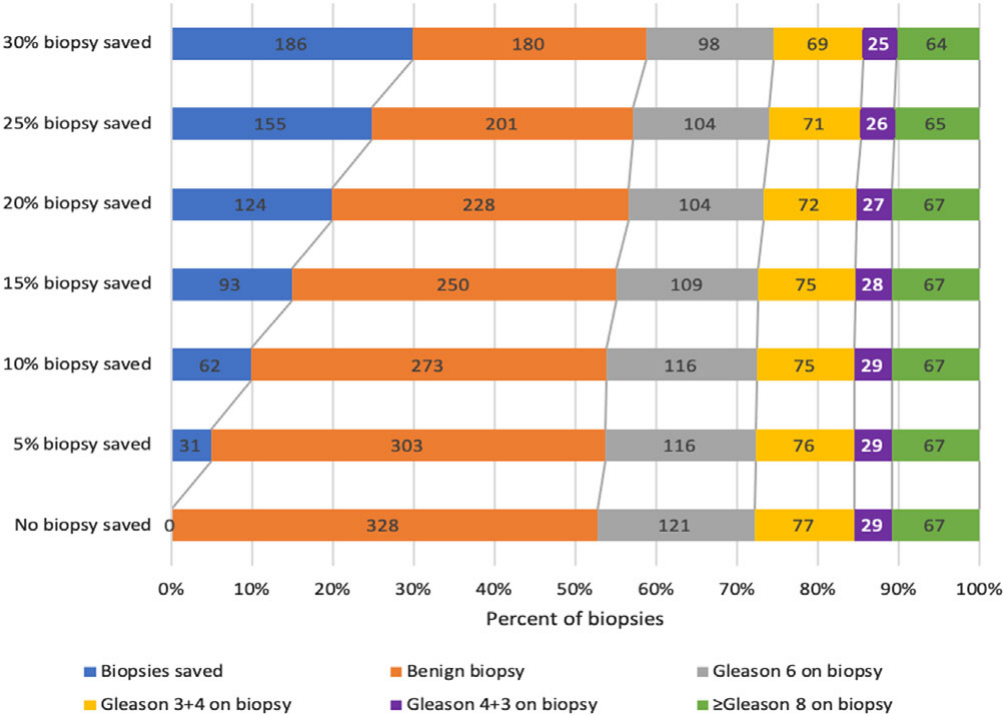
Graph showing the number of biopsies that can be saved in the validation cohort using the prediction tool predicting clinically significant prostate cancer (csPCa)

Using our model in the validation cohort, 10% of biopsies could be avoided without missing uPCa and with missing 1% csPCa, avoiding 17% of benign biopsies and avoiding 4% of clinically indolent PCa (Figure 5). Additionally, 15%, 20%, 25%, and 30% of biopsies could be avoided while missing 2%, 4%, 6%, and 9% of csPCa, respectively, avoiding 24%, 31%, 39%, and 41% of benign biopsies, respectively, and avoiding 10%, 14%, 14%, and 19% of clinically indolent PCa, respectively.

## 4 DISCUSSION

Multiparametric MRI and the 4K score test are both used in American clinical practice for the evaluation of prostate cancer. To our knowledge, this is the first study investigating the use of both the 4K score test and mpMRI in combination to detect PCa, csPCa, and uPCa. Our model confers three key benefits. (a) It reduces number of biopsies without compromising detection of csPCa and uPCa rates, (b) the model shows efficacy of 4K score test and mpMRI PI‐RADS score, for predicting PCa, csPCa, and uPCa, and (c) the model showed accuracy in predicting any PCa, csPCa, and uPCa in entirely different cohort across all ranges of probabilities.

Combining both the 4Kscore and mpMRI in this newly developed model has the ability to guide prostate cancer diagnostics with more accuracy and further decrease number of unnecessary biopsies as well as the detection of non‐csPCa. By reducing the number of unneeded biopsies, patients can avoid the risk of biopsy‐related complications including pain, bleeding, and infections, and in 1%‐2% of biopsied men, life‐threatening urosepsis.^12^ Moreover, overdetection of non‐csPCa is known to cause overtreatment of indolent disease and decreased QoL from patients living with an untreated cancer.^13^, ^14^ The advantages of this novel nomogram in men pre‐PCa diagnosis are clear, but it may also have clinical value in the increasingly large group of men with low‐risk prostate cancer who are in active surveillance (AS) programs.

Studies have shown that the 4K score can improve the diagnostic discrimination of csPCa, reducing the number of required prostate biopsies. It has been suggested it could play an important clinical role in the decision‐making process prior to proceeding with initial prostate biopsy in men with an elevated PSA level or abnormal digital rectal examination (DRE) or after a prior negative biopsy and persistently abnormal PSA levels.^15^ The recent prospective US validation study showed that 4K score test can predict csPCa with AUC 0.82 and with excellent calibration.^11^ In the French arm of the ERSPC (European Randomized Study of Prostate Cancer Screening), AUC for detecting csPCa increased from 0.77 for a basic model (age, total PSA, and DRE) to 0.87 after adding four‐kallikrein panel.^16^ Our study confirms significant role of 4K score test in predicting csPCa. Additionally, multiple studies have shown that mpMRI helps in identifying a higher proportion of csPCa when compared to transrectal ultrasound‐guided prostate biopsies alone. In the large multicenter, paired‐cohort study, PROMIS, (comparing the diagnostic accuracy of mpMRI and TRUS‐biopsy against template prostate mapping biopsy), results show that mpMRI and targeted biopsies detected up to 18% more cases of csPCa compared with TRUS‐biopsy for all, while avoiding diagnosis of nonsignificant PCa by 5%.^8^ However, other studies have shown a wide range of sensitivity for detecting csPCa (44%‐87%) as well as negative predictive values ranging from 63%‐98%.^7^

Probabilistic estimates and predictions are being used with increasing frequency in prostate cancer research to guide the decision‐making process. Huge data sets and increasingly sophisticated statistical software allow for individualized probability predictions. However, these predictions can be only helpful if they accurately reflect the correct underlying probabilities.^17^ Calibration of the prediction model is a critical component of its accuracy and thereby its clinical utility and more effective individualized care. The feature of this prediction tool is that it has been calibrated in an entirely different cohort and showed accuracy between predicted and actual rates of PCa, csPCa, and uPCa across all ranges probability percentages. Approximately, 70% of patients that undergo prostate biopsy has negative results.^18^ History of negative prostate biopsy in the past lowers the chances of finding PCa in the forthcoming prostate biopsy.^18^ Finding the cancer at earlier stage is equally important for oncological efficacy as well as addressing quality‐of‐life issues after the surgery.^19^ We found significance of prior negative biopsy for predicting PCa, csPCa, and uPCa.

Our study has some limitations. First, all biopsies were performed by a single experienced, high‐volume expert, which could affect reproducibility. Second, as mentioned in methodology, not all men who had 4K score test done were included in the analysis. Stringent biopsy criteria could also affect generalizability.

## 5 CONCLUSIONS

We have developed a 4K score/MRI‐based tool to assist clinicians in biopsy decision‐making and counselling of men at risk for PCa. Using our novel prediction model could significantly reduce the large number of biopsies that detect benign or clinically insignificant PCa, while missing only a small proportion of csPCa and uPCa. Our results demonstrate the importance of combining 4K score test, prior negative biopsy, prostate volume, and mpMRI PI‐RADS score ≥4 for predicting PCa, csPCa, and uPCa. This novel model could reduce unnecessary biopsies and decrease detection of clinically insignificant prostate cancer more effectively than mpMRI or 4K score alone. Thus, this model could have a significant impact on patient morbidity and social economic costs within prostate cancer diagnostics.

## CONFLICT OF INTEREST

The authors declare no conflicts of interest.

## AUTHOR CONTRIBUTIONS

*Conceptualization, Data curation, Formal analysis, Methodology, Project administration, Visualization, Writing‐original draft, Writing‐review and editing*, V.W.; *Data curation, Software, Writing‐review and editing*, S.S.; *Data curation, Formal analysis, Methodology*, P.R.; *Investigation, Resources, Writing‐review and editing*, J.Y.; *Investigation, Writing‐review and editing*, A.L.;. *Data curation, Investigation, Writing‐review and editing*, S.P.; *Investigation, Writing‐review and editing*, U.F. and S.L.; *Data curation, Investigation*, L.L.; *Data curation, Writing‐review and editing*, K.H.; *Validation, Writing‐review and editing*, S.P.; Conceptualization, Writing‐original draft, Writing‐review and editing, P.W.; *Conceptualization, Project administration, Resources, Supervision, Writing‐original draft, Writing‐review & editing*, A.T.

## ETHICAL STATEMENT

The study was conducted at the Icahn School of Medicine at Mount Sinai Health System (ISMMS) after approval from the Institutional Review Board (GCO 19‐1711). Informed patient consent waived for this study.

## Data Availability

The data that support the findings of this study are available from the corresponding author upon reasonable request.
